# The *lolB* gene in *Xanthomonas campestri*s pv. *campestris* is required for bacterial attachment, stress tolerance, and virulence

**DOI:** 10.1186/s12866-021-02416-7

**Published:** 2022-01-07

**Authors:** Chao-Tsai Liao, Chih-En Li, Hsiao-Ching Chang, Chien-Hui Hsu, Ying-Chuan Chiang, Yi-Min Hsiao

**Affiliations:** Department of Medical Laboratory Science and Biotechnology, Central Taiwan University of Science and Technology, Taichung, Taiwan

**Keywords:** *Xanthomonas campestris*, Stress tolerance, Virulence

## Abstract

**Background:**

*Xanthomonas campestris* pv. *campestris* (*Xcc*) is a Gram-negative bacterium that can cause black rot disease in crucifers. The lipoprotein outer membrane localization (Lol) system is involved in the lipoprotein sorting to the outer membrane. Although *Xcc* has a set of annotated *lol* genes, there is still little known about the physiological role in this phytopathogen. In this study, we aimed to characterize the role of LolB of *Xcc* in bacterial attachment, stress tolerance, and virulence.

**Results:**

To characterize the role of LolB, *lolB* mutant was constructed and phenotypic evaluation was performed. The *lolB* mutant revealed reductions in bacterial attachment, extracellular enzyme production, and virulence. Mutation of *lolB* also resulted in reduced tolerance to a myriad of stresses, including heat and a range of membrane-perturbing agents. Trans-complementation of *lolB* mutant with intact *lolB* gene reverted these altered phenotypes to the wild-type levels. From subsequent reporter assay and reverse transcription quantitative real-time polymerase chain reaction (RT-qPCR) analysis, the expression of genes that encode the major extracellular enzymes and the stress-related proteins was reduced after *lolB* mutation.

**Conclusions:**

The results in this work contribute to the functional understanding of *lolB* in *Xanthomonas* for the first time, and provide new insights into the function of *lolB* in bacteria.

## Background

In Gram-negative bacteria, the outer membrane presents a selectively permeable barrier to the environment and is the first line of defense against harmful chemicals, including detergents and antibiotics [1]. Bacterial lipoproteins are a set of membrane proteins localized on either leaflet of the lipid bilayer and are important components of the Gram-negative cell envelope [2, 3]. In *Escherichia coli*, most lipoproteins are considered to be anchored to the inner leaflet of the outer membrane [3, 4]. The Lol (lipoprotein outer membrane localization) pathway is responsible for sorting and localizing lipoprotein [2–5].

The Lol pathway has components in each compartment of the cell envelope: an ATP binding cassette transporter LolCDE in the inner membrane; a soluble chaperone protein LolA in the periplasmic space; and a lipoprotein LolB in the outer membrane [4]. The outer membrane-directed lipoprotein is extracted from the inner membrane by LolCDE, transferred to LolA, and shuttled to the outer membrane, where LolB receives and then anchors them into the bilayer [2–5]. The Lol proteins of *E. coli* have been studied in depth and each of the *lol* genes are considered to be essential for viability of this bacterium [2, 3, 5].

The Lol homologues can be found in many Gram-negative bacteria, suggesting that the pathway is conserved [2, 3, 5]. However, conservation of individual Lol protein encoding genes varies. In most γ-proteobacteria, the LolCDE consists of one copy each of membrane subunits LolC and LolE, and two copies of LolD [3]. LolC and LolE are homologues but cannot functionally substitute each other in *E. coli* [6]. However, some bacterial genomes contain only one copy of a *lolC/lolE* gene; the protein product contains sequence motifs of both LolC and LolE and the LolF name was proposed to distinguish such proteins from obvious LolC and LolE homologues [7]. Additionally, phylogenetic analysis suggests that *lolB* gene is only conserved in β- and γ-proteobacteria [2, 5]. Till now, only the Lol proteins of *Pseudomonas aeruginosa* have been indicated to involve in the sorting of outer membrane lipoprotein, as in the case of *E. coli* lipoproteins [8, 9]. Recently, it has been reported that the LolCDE proteins of the pathways of *E. coli* and *P. aeruginosa* are interchangeable [10].

*Xanthomonas campestris* pv. *campestris* (*Xcc*), a Gram-negative bacterium, is capable of causing cruciferous plant infections. This pathogen causes black rot disease in the members of *Brassica oleracea* such as broccoli, cabbage, cauliflower, and radish [11–13]. The virulence of *Xcc* depends on a number of factors, including biofilm formation, extracellular enzymes (such as cellulase, mannanase, and protease), and extracellular polysaccharides [14, 15]. Four *lol* genes (*lolA*, *lolB*, *lolD*, and *lolF*) have been annotated in the fully sequenced *Xcc* genome [12, 16–18]. Among them, only *lolA* has been studied. The *Xcc lolA* has been indicated to play a role in pathogenicity and stress tolerance [19]. The aims of the present work are to characterize *lolB* in *Xcc*. The role of *lolB* in bacterial attachment, extracellular enzyme production, stress tolerance, and virulence was examined in this study.

## Methods

### Bacterial strains and growth conditions

Table 1 lists the bacterial strains and plasmids used in this study. *E. coli* and *Xcc* were grown at 37 °C and 28 °C, respectively. Luria–Bertani (LB) was used as a routinely cultured medium [20]. XOLN was a basal salt medium and contained 0.625 g/L tryptone and 0.625 g/L yeast extract [21]. Glucose or glycerol was added (2%) as required. Liquid cultures were shaken at 180 rpm. Solid media contained 1.5% agar. Growth media was supplemented with antibiotics when required. The added antibiotics and concentrations are: ampicillin (50 μg/mL), gentamycin (15 μg/mL), kanamycin (50 μg/mL), and tetracycline (15 μg/mL).

**Table 1:** Bacterial strains and plasmids used in this study

Strain or plasmid	Description	Reference or source
***E. coli***		
ECOS™ 101	*endA1 recA1 relA1 gyrA96 hsdR17*(*r*_K_^−^, *m*_K_^+^) *phoA supE44 thi-1* Δ(*lacZYA-argF*) *U169* ϕ80Δ*(IacZ*)M15 F^−^	Yeastern
***X. campestris* pv. *campestris***		
XC17	Virulent wild type strain isolated in Taiwan, Ap^R^	[22]
CL17	XC17-derived mutant with EZ-Tn*5* inserted in *lolB* gene, Ap^R^, Km^R^	This study
**Plasmid**		
yT&A	PCR cloning vector, Ap^R^	Yeastern
pTlolB	A 790 bp RCR amplified fragment from *lolB* (nucleotides –133 to + 657 relative to the translation start site) and cloned into yT&A	This study
pUC19G	Gm^R^ cartridge from pUCGM ligated with the blunt-ended *Ava*II-*Ssp*I large fragment from pUC19	[23]
pUClolB	The 790 bp *Bam*HI-*Eco*RI fragment of the pTlolB cloned into the *Bam*HI and *Eco*RI sites of pUC19G	This study
pUClolBK	pUClolB derivative with Km^R^ inserted in the internal region of *lolB* gene	This study
pRK415	Broad-host-range vector, RK2 *ori*, Tc^R^	[24]
pRKlolB	The 790 bp *Bam*HI-*Eco*RI fragment of the pTlolB cloned into the *Bam*HI and *Eco*RI sites of pRK415	This study
pFY13–9	Promoter-probing vector derived from pRK415, using *lacZ* as the reporter, Tc^R^	[25]
pFYengA	The 159-bp fragment, –181/–23 relative to *engA* translation start site, cloned into the *Pst*I/*Xba*I sites of pFY13-9	This study
pFYmanA	The 360-bp fragment, –372/–13 relative to *manA* translation start site, cloned into the *Pst*I/*Xba*I sites of pFY13-9	This study
pFYprt1	The 313-bp fragment, –392/–80 relative to *prt1* translation start site, cloned into the *Pst*I/*Xba*I sites of pFY13-9	[26]
pFYclpP1	The 375-bp fragment, –384/–10 relative to *clpP* translation start site, cloned into the *Xho*I/*Xba*I sites of pFY13-9	[27]
pFYclpX	The 327-bp fragment, –336/–10 relative to *clpX* translation start site, cloned into the *Xho*I/*Xba*I sites of pFY13-9	This study

Ap^R^, ampicillin-resistant; Gm^R^, gentamycin-resistant; Km^R^, kanamycin-resistant; Tc^R^, tetracycline-resistant

### Recombinant DNA techniques

Bacterial genomic DNA and plasmid DNA were purified using the Wizard® Genomic DNA Purification Kit (Promega) and the Gene-Spin™ Miniprep Purification Kit (Protech), respectively. Polymerase chain reaction (PCR) was carried out as previously described [28]. Table 2 lists the primers used in this study. Standard protocols for agarose gel electrophoresis, DNA ligation, restriction digestion, and *E. coli* transformation were as described previously [29]. Transformation of *Xcc* was achieved through electroporation [30]. The sequence of DNA fragment was determined by Mission Biotech Co., Ltd. (Taipei, Taiwan).

**Table 2:** Primers used in this study

Primer	Sequence (5’ to 3’) ^***a***^
***lolB* gene (mutant construction, confirmation and complementation)**	
lolBF/lolBR	GGATCCAAATCGCCGCGCACGTGGGT/GAATTCAGGGCGAGAGCGTCCATTGG
**Extracellular enzyme encoding genes (promoter analysis and RT-qPCR)**	
***engA* gene**	
844pstF/1002xbaR	AACTGCAGCCTGCGGACAGCGCGCAGGGGG/GCTCTAGAGCTCGACACCCGAGCGCGGTAA
engAF/engAR	GTGTGAACGTGTTCGGCTTC/TCATGTCCTTCCAGTTGCGT
***manA* gene**	
61pstF/420xbaR	CTGCAGTTGGCCGCGCATGCGA /TCTAGAACA AGGTGG ACGCCGCAGAC
manAF/manAR	AGTTCTACATGCGCGACAAC/CGTACATGTGCACGCTGAAA
***prt1* gene**	
200pstF/512xbaR	AACTGCAGTGTCGCTGCGCCAGGAGCTGAC/GCTCTAGACGGATCGCCCCTGTTATCGATC
prt1F/prt1R	CACCGCACAGACCCATCAGA/TTACCAGTTCCGGCCCCAAC
**Stress tolerance related genes (promoter analysis and RT-qPCR)**	
***clpP* gene**	
1315XhoI/1689XbaI	CTCGAGGGGTTCATGGACGCCGCT/TCTAGATGTTGTGGCAGCGGCCTGTG
clpPF/clpPR	AGATCCTGACCTTGCGTTCG/CTTGAAGTTGTCGCGTTCGG
***clpX* gene**	
114xhoF/440xbaR	CTCGAGGCGACCGATATCGACATCCA/TCTAGACCCAGTTACCCCACCCGATG
clpXF/ clpXR	CTCGAGGAACTCGATGAGCC/AGCTCCACGCTTTCCATCTC
**Putative lipoprotein encoding genes (RT-qPCR)**	
0253F/0253R	GCAATTACCAGCTGCGCTAC/TTGGTGACATCCTCGAACGG
0677F/0677R	GGCGACTTCAATTGCTACCG/GCACCAGTTTCCATAACGCC
0679F/0679R	GCCGATTTCAACAAGGCCAA/TTCGTCGTTGGGAAAGGTCT
0707F/0707 R	GGGTCATCGACCTGAGCTAC/CGCGTACCTCGACATTACCG
1519F/1519R	GCCTACGTGTGGAACGAACA/CGGTCATGGTCGAACTGCAT
1584F/1584R	CGACAGACGCTGTACGAAGA/TATTGACGCGTGCAAAGTGC
3831F/3831R	TGAAGATCCACTGGGCCGTA/TTCGGGTTTCTGCTCGGTC
4152F/4152R	CGCAATGTGCCATTGGTGAT/CTCCGTGGTATCGAACAGGC
***16S rRNA* gene (RT-qPCR)**	
16SF/16SR	GTAAAGCGTGCGTAGGTGGT/CGTGCCTCAGTGTCAGTGT

^***a***^**:** Added restriction enzyme sites are underlined

### *lolB* mutant construction and complementation

For the construction of *lolB* mutant, the 790-bp *Bam*HI-*Eco*RI fragment containing the upstream 133-bp and the entire coding region of the XC17 *lolB* was PCR-amplified using primers lolBF/lolBR and cloned into the yT&A (Yeastern) to produce pTlolB. After sequence verification, the fragment was excised and cloned into the *Bam*HI-*Eco*RI sites of pUC19G [23] to generate pUClolB. The EZ-Tn*5*™ < KAN-2 > Transposon (Km^R^, 1221 bp) was randomly inserted into pUClolB using the EZ-Tn*5*™ < KAN-2 > insertion kit according to the manufacturer’s instructions (Lucigen). One plasmid, pUClolBK, with the transposon inserted into the *lolB*-coding sequence at 326 bp from the translational start site was used for mutant construction. This plasmid was then introduced into the *Xcc* wild-type XC17 by electroporation, allowing for double crossover, and transformants were selected on LB medium supplemented with kanamycin (transposon selection marker). Insertion of transposon into *lolB* gene was confirmed by PCR. The confirmed *lolB* mutant was designated as CL17.

For complementation of the *lolB* mutant, the 790-bp *Bam*HI-*Eco*RI fragment of pTlolB was excised and inserted into the *Bam*HI-*Eco*RI sites of pRK415 [24]. The generated plasmid pRKlolB was transferred into the *lolB* mutant strain CL17 by electroporation, giving the complemented strain CL17(pRKlolB). For phenotypic comparison, the empty vector pRK415 was introduced into XC17 and CL17, giving transformants XC17(pRK415) and CL17(pRK415) in parallel.

### Assays of bacterial attachment and pathogenicity

The bacterial attachment was evaluated by examining the ability of cells to adhere to the 96-well polystyrene microtiter plates (Nunc) and cabbage leaves surface as the previously described method [31]. The experiments were done at least three times. The pathogenicity of *Xcc* to host plant cabbage was tested by leaf-clipping method [26] and the disease symptoms in cabbage were photographed and lesion lengths were measured 14 days after inoculation. Testing was performed in three independent experiments, each with six replicates.

### Extracellular enzyme activity analysis

Extracellular enzyme activity was analyzed by spotting 3 μL of overnight culture (OD_550_ = 1) onto XOLN agar plates containing the appropriate substrates. The substrates used are: carboxymethyl cellulose (0.5%, substrate for cellulase), locus bean gum (0.2%, for mannanase), and skimmed milk (1%, for protease). After 2 days (cellulase and mannanase) or 3 days (protease) of incubation, enzyme activity was determined as described previously [32]. Each test was carried out at least three replicates.

### Stress tolerance assay

Stress tolerance was tested by inoculating overnight culture into fresh XOLN medium containing glycerol to obtain an initial OD_550_ of 0.35 in the absence or presence one type of stress condition. The stresses and their concentrations used were as following: EDTA (0.2 mM), H_2_O_2_ (0.005%), polymyxin B (2 μg/mL), puromycin (10 μg/mL), and sodium dodecyl sulfate (SDS, 0.0075%). The growth of each strain was determined by measuring the OD_550_ values after incubation with shaking (180 rpm) at 28 °C for 24 h. The method for temperature tolerance assay was according to previously study [31]. Each stress test was repeated at least three times.

### Cell membrane integrity analysis

The integrity of *Xcc* cell membrane was examined by the SYBR Green I/propidium iodide (PI) viability assay as the previously described methods [33, 34] with some modifications. Briefly, the cultured bacteria were harvested by centrifuging at 12,000 rpm for 2 min and washed twice then resuspended in sterile 0.85% NaCl. The final cell suspension was adjusted to an OD_550_ = 1. Then, the bacterial cells (100 μL) were stained with SYBR Green I (2X, 100 μL) and PI (250 mg/mL, 10 μL). The samples were incubated for 40 min in dark at room temperature. After staining, the samples were washed twice and resuspended in 50 μl 0.85% NaCl, and 5 μl of this sample was trapped in between coverslip and glass slide. The slide was viewed under a fluorescence microscope.

### Reporter plasmid construction and promoter activity analysis

Reporter constructs (pFYengA, pFYmanA, and pFYclpX) were generated by cloning the PCR-amplified upstream regions of *engA*, *manA*, and *clpX* into pFY13–9 [25], with *lacZ* as the reporter. Briefly, the upstream region of each gene was amplified by PCR using primers 844pstF/1002xbaR for the *engA* gene, 61pstF/420xbaR for *manA* gene, and 114xhoF/440xbaR for the *clpX* gene. Then, the PCR fragments were cloned into pFY13–9, giving rise to pFYengA, pFYmanA, and pFYclpX. Reporter constructs pFYprt1 and pFYclpP1 containing the upstream regions of *prt1* and *clpP*, respectively, were obtained as previously described [26, 27]. *Xcc* strains harboring these constructs were grown overnight and inoculated into fresh media to obtain an initial OD_550_ of 0.35, after which growth was allowed to continue. Samples were taken in triplicate at designated intervals and the β-galactosidase activity was assayed as previously described, with the enzyme activity expressed in Miller units [20].

### RNA isolation, reverse transcription (RT), and quantitative real-time PCR (qPCR)

Total RNA was isolated from bacteria grown to the mid-exponential phase (OD_550_ = 0.6) in XOLN medium supplemented with 2% glycerol using the RNeasy Mini Kit (Qiagen) according to provided instructions. The isolated RNA (1 μg) was reverse-transcribed to cDNA using the iScript™ gDNA Clear cDNA Synthesis Kit (BIO-RAD). qPCR was performed using iQ™ SYBR® Green Supermix in a CFX96 Real Time PCR system (BIO-RAD). The PCR amplification conditions were as follows: 3 min at 95 °C, followed by 40 cycles of 10 s at 95 ^º^C, 10 s at 60 ^º^C, and 30 s at 72 ^º^C. Table 2 lists the sequences of primer sets of the tested target genes. The *Xcc 16S rRNA* gene was used for normalization. All qPCRs were performed at least three times. The fold change for transcript was calculated by the 2^−ΔΔ*Ct*^ method.

### Statistical analysis

Each experiment was carried out at least three repeats. Values are the averages of three replications per experiment. Student’s *t* test was used to evaluate the statistical significance of differences between averages. A *p* value < 0.05 was considered statistically significant.

## Results

### Disruption of *lolB* leads to decrease bacterial attachment

In the genome of *Xcc* strain XC17, the locus_tag AAW18_RS04315 is annotated to encode lipoprotein insertase outer membrane protein LolB (Gen-Bank accession no. NZ_CP011946) [16]. The XC17 *lolB* open reading frame is 657 bp in length and is located in the genome sequence at positions 1,003,373–1,004,029 [16]. The XC17 *lolB* gene are found in several sequenced *Xcc* strains, such as ATCC33913, 8004, and B100 [12, 17, 18]. Through sequence comparison, it was found that the coding product of XC17 *lolB* was identical in both amino acid sequence and size to LolBs from *Xcc* strains ATCC33913 and 8004. The orthologous gene of *lolB* was also highly conserved in other members of *Xanthomonas*, such as *X. campestris* pv. *raphani* 756C [35], *X. campestris* pv. *vesicatoria* 85–10 [36], *X. citri* subsp. *citri* (formerly *X. axonopodis* pv. *citri*) 306 [18], *X. hortorum* pv. *gardneri* ICMP 7383 [37], and *X. oryzae* pv. *oryzae* KACC10331 [38] (Table 3). Although the *lolB* gene has been found in several members of *Xanthomonas*, none of them has been characterized with regard to function, and no relevant studies were found in the literature.

**Table 3:** LolB homologues in *Xanthomonas* spp

Bacteria^**a**^	Gene ID	Predicted product	Size (aa)	Identities (%)^**b**^
*X. campestris* pv. *campestris* ATCC33913	XCC0870	Outer membrane lipoprotein precursor	218	100
*X. campestris* pv. *campestris* 8004	XC_3360	Outer membrane lipoprotein precursor	218	100
*X. campestris* pv. *campestris* B100	Xccb100_3479	Outer membrane lipoprotein receptor LolB	218	99.1
*X. campestris* pv. *raphani* 756C	XCR_1061	Outer membrane lipoprotein LolB	218	98.6
*X. campestris* pv. *vesicatoria* 85–10	XCV0978	Outer membrane lipoprotein receptor LolB	217	84.4
*X. axonopodis* pv. *citri* 306	XAC0947	Outer membrane lipoprotein precursor	217	85.3
*X. hortorum* pv. *gardneri* ICMP 7383	BI317_05530	Lipoprotein localization factor LolB	218	85.6
*X. oryzae* pv. *oryzae* KACC10331	XOO3605	Outer membrane lipoprotein precursor	217	84.4

^**a**^: *X. campestris* pv. *campestris* ATCC33913 (GenBank accession number: AE008922); *X. campestris* pv. *campestris* 8004 (CP000050); *X. campestris* pv. *campestris* B100 (AM920689); *X. campestris* pv. *raphani* 756C (CP002789); *X. axonopodis* pv. *citri* 306 (AE008923); *X. hortorum* pv. *gardneri* ICMP 7383 (CP018731); *X. campestris* pv. *vesicatoria* 85–10 (AM039952); *X. oryzae* pv. *oryzae* KACC10331 (AE013598)

^**b**^: According to a BLASTP search

**Fig. 1:**
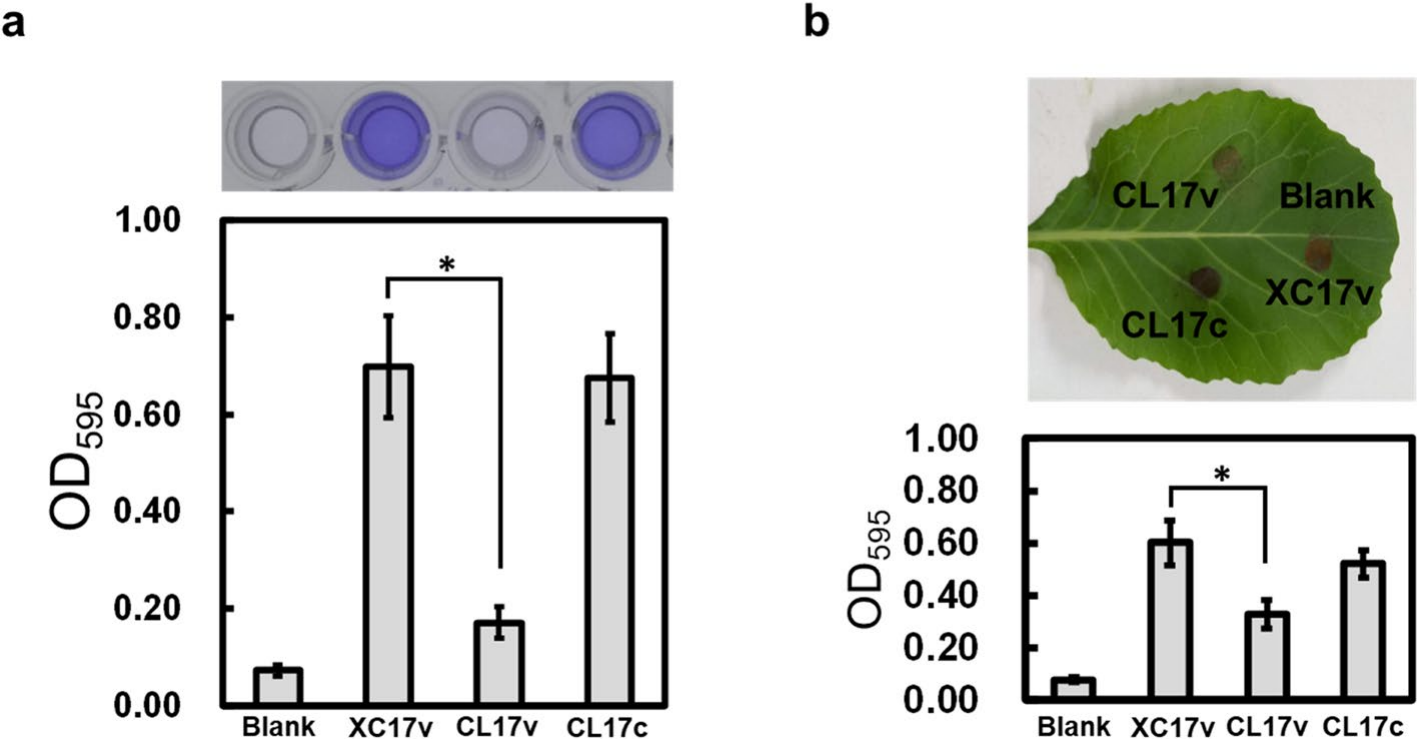
Effects of mutation of *lolB* on cell attachment to polystyrene plates (**a**) and cabbage leaf surfaces (**b**) in *Xcc*. Strains to be tested were grown overnight, washed, and diluted using fresh XOLN medium supplemented with glucose, and were assayed as described in the Material and methods section. XC17v: wild-type strain XC17 carrying empty vector pRK415; CL17v: *lolB* mutant CL17 carrying pRK415; CL17c: complemented *lolB* mutant; Blank: XOLN medium supplemented with glucose without inoculation of bacteria. Values presented are the mean ± standard deviation (*n* = 3). Significance was determined using the Student *t* test (* indicates significance at *p* < 0.05)

To explore the physiological role of *lolB* in *Xcc*, the *lolB* mutant and its complemented strain were generated. Biofilm formation was tested on polystyrene microtiter plate (Fig. 1a), and leaf surface (Fig. 1b). As depicted in Fig. 1, it was indicated that the *lolB* mutant exhibited reduced attachment ability compared with the parental strain, and complementation of *lolB* mutant with plasmid pRKlolB (with intact *lolB* gene cloned in pRK415) could restore the adhesion ability to the wild-type level.

### The *lolB* gene is required for the full virulence of *Xcc*

**Fig. 2:**
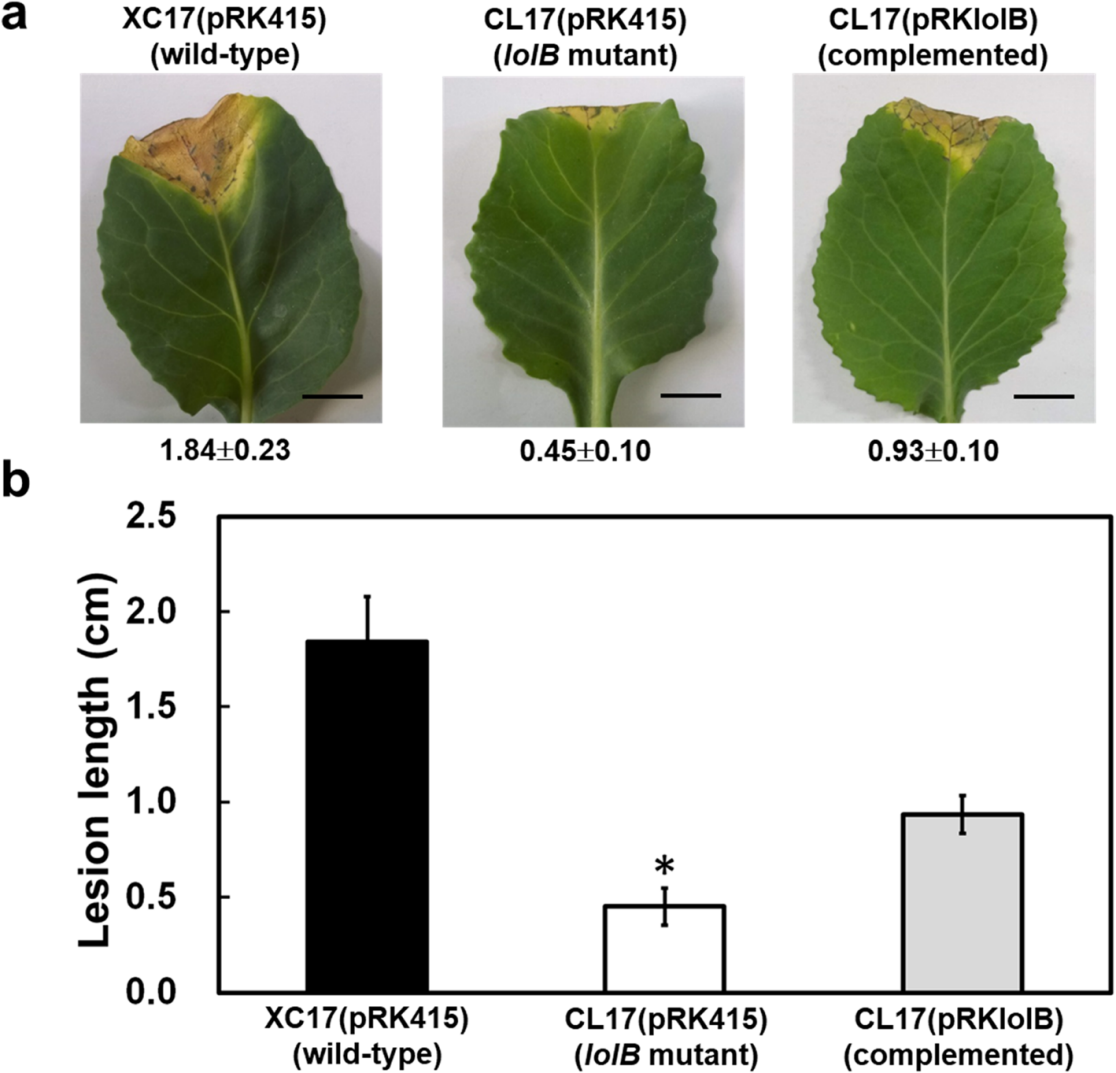
Effects of mutation of *lolB* on virulence of *Xcc* in cabbage. (**a**) Black rot symptoms caused by *Xcc* strains on inoculated leaves of host cabbage plant. After 14 days inoculation, the photographs were taken. (**b**) Mean lesion lengths caused by different *Xcc* strains. Values shown are the average ± standard deviation from three repeats, each with six leaves. Significance was determined using the Student *t* test (* indicates significance at *p* < 0.05)

To determine whether mutation of *lolB* caused loss of pathogenicity, *lolB* mutant was used to infect host plant cabbage by leaf-clipping method. At 14 days post inoculation, typical V-shaped black rot symptoms were found on leaves inoculated with the wild-type strain (Fig. 2a) and the lesion lengths were about 1.84 cm (Fig. 2b). However, the *lolB* mutant shown reduced virulence compared with the wild-type strain (Fig. 2a) and the lesion lengths caused by the mutant were about 0.45 cm (Fig. 2b). The complementation of *lolB* mutant with pRKlolB partially restored the virulence of the mutant (Fig. 2a). Although the complementary strain cannot fully restore pathogenicity, its consequent mean lesion length (0.93 cm) was significantly longer than that inoculated with the mutant strain (Fig. 2b). These results indicated that *lolB* is important for host virulence of *Xcc*.

### The *lolB* gene is involved in extracellular enzyme production

**Fig. 3:**
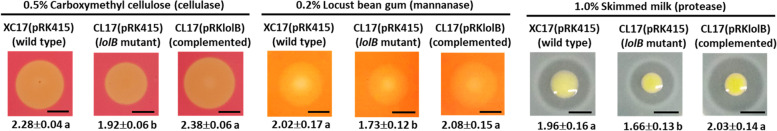
Effects of mutation of *lolB* on extracellular enzyme production in *Xcc*. The extracellular enzyme activity was evaluated using the substrate-supplemented plate assay as described in the Material and methods section. Values under each photographs are the diameter of hydrolysis zone (in cm) (mean ± standard deviation) from three independent experiments. Different letters following the values indicate significant difference (Student *t* test, *p* < 0.05)

It has been indicated that extracellular enzymes and extracellular polysaccharides contribute to the virulence of *Xcc* [39, 40]. The reduced virulence of the *lolB* mutant (Fig. 2) suggested that the *lolB* gene has roles in the production of these pathogenicity-related determinants. The activity of extracellular hydrolytic enzymes (including cellulase, mannanase, and protease) was first tested. The results showed that the levels of extracellular cellulase and mannanase were reduced in the *lolB* mutant and could be restored by complementation (Fig. 3). In the protease assays, the diameters of the hydrolysis zones formed by the *lolB* mutant were significantly smaller than those found for the wild type and complementary strains. As the colony diameter of wild type are larger than those of *lolB* mutant and the complementary strain, *lolB* might be not involved in protease production, although small effects could not be excluded. Next, extracellular polysaccharide production was tested. The extracellular polysaccharide yields produced by the mutant were similar to those of the wild type (data not shown).

### The *lolB* mutant displays increased sensitivity to various stresses

**Fig. 4:**
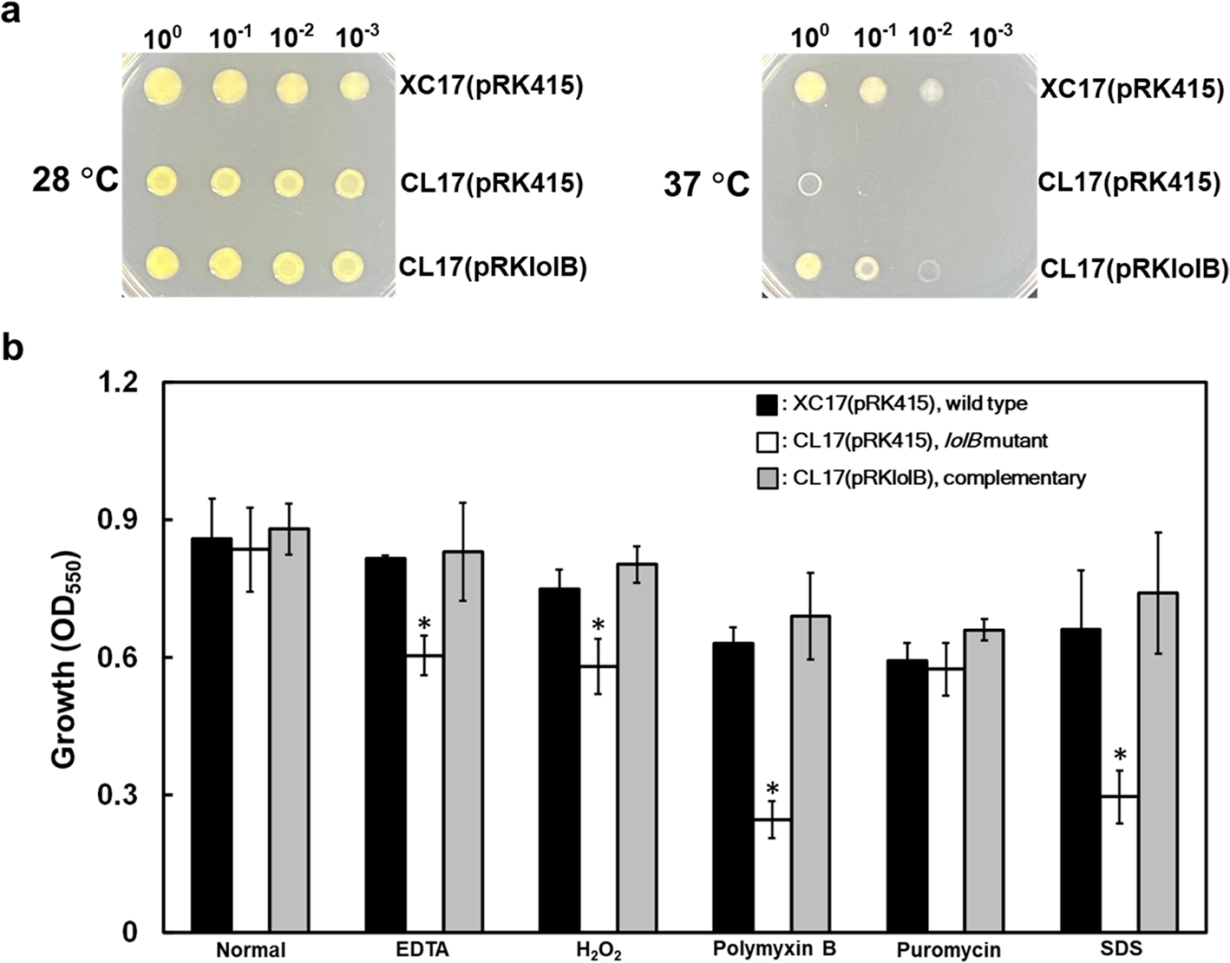
Effects of mutation of *lolB* on stress tolerance. (**a**) Heat tolerance was carried out with tenfold dilution of cells spotted on LB plate and incubated at 28 °C or 37 °C for 3 days. (**b**) The effects of a range of chemicals on bacterial growth were determined quantitatively in liquid culture. Bacteria cells were grown in XOLN medium with or without different stresses. After 24 h, the cell density was determined at OD_550_. Values shown are the averages ± standard deviations from three repeats. The asterisk (*) indicates *p* < 0.05

As several factors reported to influence bacterial attachment also have roles in stress tolerance in *Xcc* [19, 31, 41, 42], we aimed to determine whether the *lolB* gene was required for stress adaptation of *Xcc*. To examine whether *lolB* contributes to stress tolerance, the sensitivity of the *lolB* mutant together with the wild-type and complementary strains was evaluated under a range of stresses, including heat, EDTA, H_2_O_2_, polymyxin B, puromycin, and SDS. At physiological temperature (28 ^º^C), the tested strains plated at all densities grew normally (Fig. 4a, left). When bacterial strains grew at elevated temperature (37 ^º^C), the growth of *lolB* mutant was inhibited and this grow deficiency was restored by genetic complementation (Fig. 4a, right). When bacterial strains were exposed to EDTA, H_2_O_2_, polymyxin B, and SDS, the *lolB* mutant exhibited significant growth reduction compared to the wild type and complementary strains (Fig. 4b). These data indicated that *lolB* is involved in stress in *Xcc*.

### Mutation of *lolB* influences the expression of genes encoding extracellular enzymes and involved in stress tolerance

**Fig. 5:**
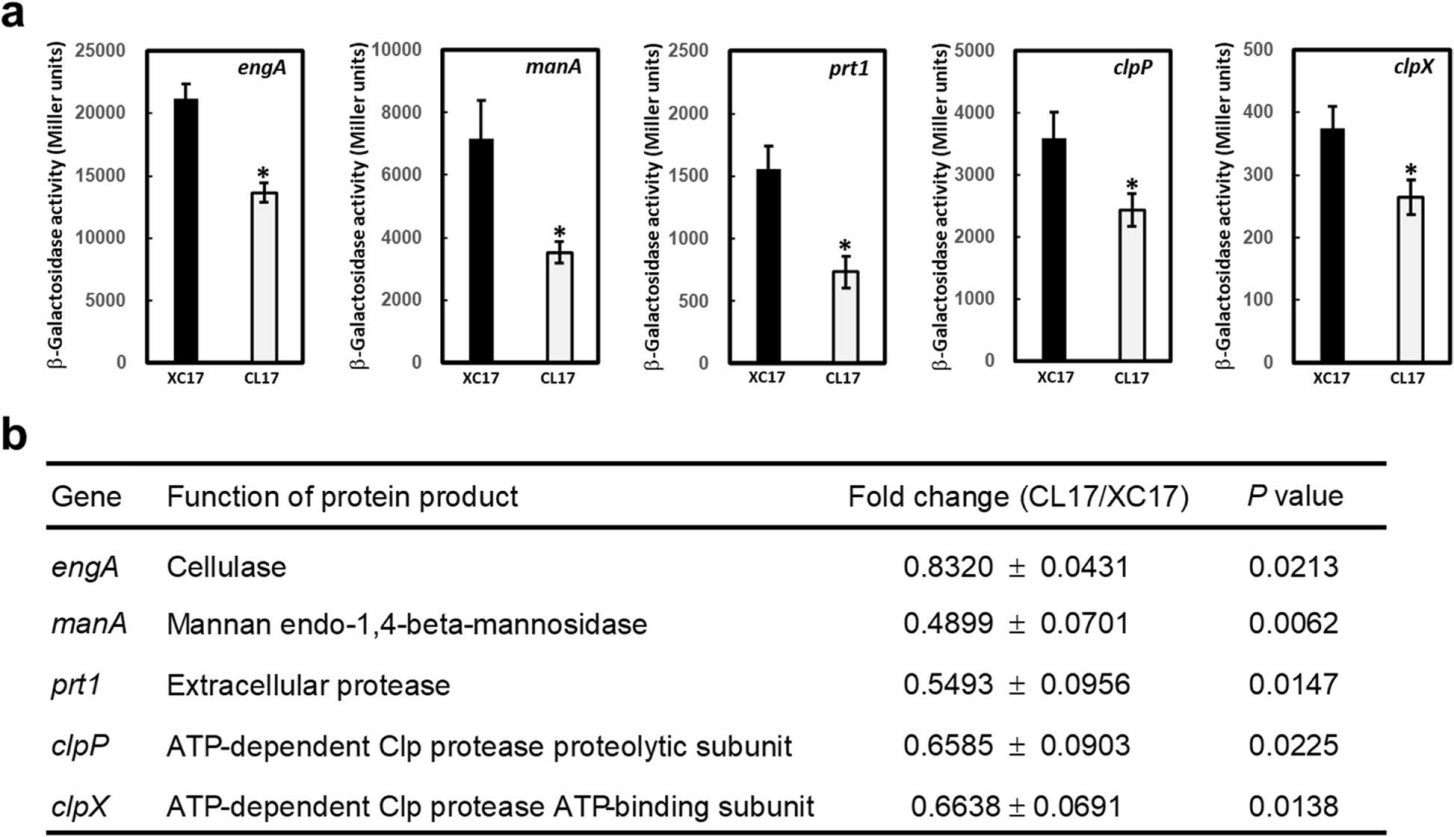
Effects of mutation of *lolB* on the expression of genes coding for extracellular enzymes or products associated with stress tolerance. (**a**) ß Galactosidase activities of XC17 (wild type, black bar) and CL17 (*lolB* mutant, gray bar) carrying pFYengA, pFYmanA, pFYprt1, pFYclpP1 and pFYclpX were determined. Tested strains were cultured in XOLN medium containing glycerol for 24 h. Significance was determined by the Student t test (* indicates significance at *p* < 0.05). (**b**) The expression level of extracellular enzyme genes (*engA*, *manA*, and *prt1*) and stress tolerance-related genes (*clpP* and *clpX*) in the wild-type strain XC17 and *lolB* mutant strain CL17 was examined by RT-qPCR. The relative expression level of each test gene in XC17 and CL17 was normalized to its 16S rRNA content. Values shown are the average ± standard deviation (*n* = 3)

Since mutation of *lolB* leads to reductions in bacterial attachment, extracellular enzyme production, and stress tolerance, *lolB* might be involved in expression of genes related to these phenotypes. Five genes (*engA*, *manA*, *prt1*, *clpP*, and *clpX*) were selected based on the alternated mutant phenotypes mentioned above. Among them, *engA* (encodes major cellulase) [28, 40], *manA* (encodes major mannanase) [39, 43], and *prt1* (encodes major protease) [40, 44] have been implicated as virulence factors. Both *clpP* and *clpX* (encode the proteolytic core and ATP-binding subunit of Clp protease, respectively) were known to play a role in extracellular enzyme production, stress tolerance, and virulence [27, 31]. In addition, the *clpX* gene was also reported to be involved in bacterial attachment [31]. To evaluate the involvement of *lolB* in expression of these virulence-related genes, reporter constructs containing the upstream regions of these genes (pFYengA, pFYmanA, pFYprt1, pFYclpP1, and pFYclpX) were introduced into XC17 (wild type) and CL17 (*lolB* mutant), and the resultant strains were subjected to β-galactosidase assays. As depicted in Fig. 5a, the β-galactosidase levels of CL17 harboring pFYengA, pFYmanA, pFYprt1, pFYclpP1, and pFYclpX were 64%, 49%, 47%, 68%, and 71% of the levels of XC17 carrying the same constructs. The effect of *lolB* mutation on the expression of these genes was also evaluated by RT-qPCR. The results indicated that all of the tested genes were significantly reduced in the *lolB* mutant when compared with wild type (Fig. 5b). Taken together, both sets of expression results from the reporter assay and RT-qPCR analysis suggested *lolB* mutation affects the expression of *engA*, *manA*, *prt1*, *clpP*, and *clpX*.

### Mutation of *lolB* influences the expression of genes encoding putative lipoproteins and the integrity of cell membrane

The findings showing reduced bacterial attachment and resistance to several membrane-perturbing compounds in *lolB* mutant, compared to the wild-type strain, might be due to an altered outer membrane lipoprotein profile and change in cell membrane integrity. Till now, the key amino acid residues involved in lipoprotein localization in *Xcc* remain unknown. According to DOLOP, a database of bacterial lipoproteins, 101 lipoproteins in the genome sequence of *Xcc* strain 8004 were identified [45]. To test the effects of *lolB* mutation on the expression of predicted lipoproteins, eight putative lipoprotein encoding genes are randomly selected, and the expression of these genes is evaluated by RT-qPCR. Table 4 shows that three genes, including *XC_0707*, *XC_1584*, and *XC_4152*, were not expressed differently in the *lolB* mutant and wild-type strains. However, the expression of five genes was significantly upregulated in the *lolB* mutant compared to the wild-type strain; they were genes encoding a dipeptidyl anmnopeptidase (*XC_0253*), a methanol dehydrogenase (*XC_0679*), an alkaline phosphatase (*XC_1519*), and two hypothetical proteins (*XC_0677* and *XC_3831*).

**Table 4:** Comparison of expression of putative lipoprotein encoding genes in the wild type XC17 and the *lolB* mutant CL17 by RT-qPCR

Gene ID ^**a**^	Description	Predicted lipoprotein signal ^**b**^	Fold change (CL17/XC17) ^**c**^	*p* value
XC_0253	Dipeptidyl anmnopeptidase	MQRLLLASSLLLA**LSAC**SDKS	3.1015 ± 0.1651	0.0361
XC_0677	Hypothetical protein	MKYLLSAALCVAA**LSGC**TDRE	6.9535 ± 0.4812	0.0312
XC_0679	Methanol dehydrogenase	MHQSSCRSARGGVLLMLALSAV**LAGC**KKDT	5.3956 ± 0.3450	0.0348
XC_0707	Rare lipoprotein A	MNSITGPKWLIPMALMLG**LAAC**SSAP	3.0953 ± 0.2584	0.0733
XC_1519	Alkaline phosphatase	MPMRYRLPALAALTTLC**VAAC**ASTA	1.7876 ± 0.0305	0.0177
XC_1584	Cyanoglobin	MMTRWLRYSLLCVLT**LSAC**ATTQ	3.0530 ± 0.3839	0.0615
XC_3831	Hypothetical protein	MKIHWAVLACATLA**LAAC**QRPQ	2.2654 ± 0.1078	0.0056
XC_4152	Cytochrome c biogenesis protein	MARRFPWLWLGL**LAAC**ILVA	3.4164 ± 0.3353	0.0581

^**a**^: Gene ID is based on *X. campestris* pv. *campestris* strain 8004

^**b**^: According to the DOLOP database. The predicted lipobox with invariant cysteine is bold and underlined

^**c**^: The relative expression level of each test gene in XC17 and CL17 was normalized to its 16S rRNA content. Values shown are the average ± standard deviation (*n* = 3)

**Fig. 6:**
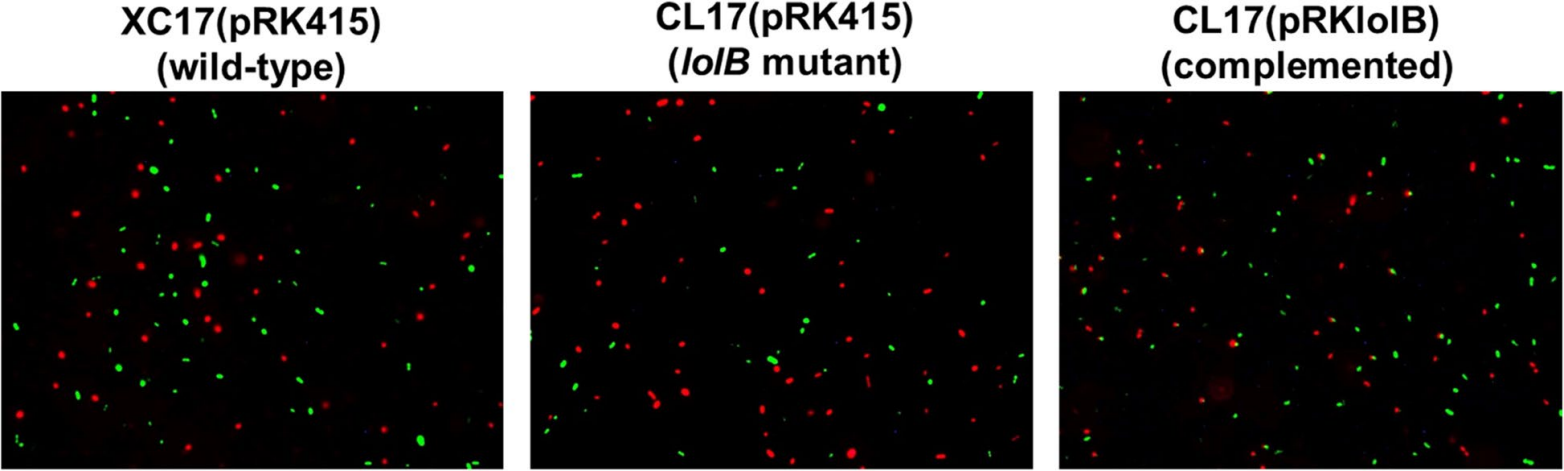
Effects of mutation of *lolB* on the integrity of cell membrane. Strains to be tested were grown overnight, washed, and diluted using 0.85% NaCl, and were stained as described in the Material and methods section. Representative microscopic images are shown. Green fluorescence represents viable cells; red fluorescence represents dead cells. Staining was performed in three independent experiments

For examining the cell membrane integrity, SYBR Green I and PI were used for double staining of nucleic acids. SYBR Green I is a green permeable dye that stains all live cells, whereas PI is a red impermeant dye that stains only dead or damaged cells with a compromised cell membrane [34]. Thus, live bacteria with intact membranes fluoresce green, while bacteria with damaged membranes fluoresce red. As shown in Fig. 6, it can be clearly seen that the wild type appeared predominantly green (indicating cells with intact membranes); whereas the *lolB* mutant appeared substantially red (demonstrating cells with damaged membranes).

## Discussion

In the fully sequenced *Xcc* genome, four *lol* genes have been annotated to encode proteins constituted to form Lol system [12, 16–18]. Till now, only *lolA* has been studied [19]. The goal of the present study was to characterize the function of *Xcc lolB*. Through genetic complementation and phenotypic evaluation, it was demonstrated that in *Xcc*, *lolB* is involved in various cellular processes, including bacterial attachment, extracellular enzyme production, pathogenesis, tolerance to a range of environmental stresses, and the maintenance of cell membrane integrity.

The LolB homologues have been found in multifarious bacteria, and there are 1435 sequences with LolB domain (Pfam03550) are listed in the Pfam family database [46]. Among them, only the LolB of *E. coli* has been characterized in detail. In *E. coli*, *lolB* is an essential gene and deletion of *lolB* is lethal and causes accumulation of lipoprotein localization intermediates in the periplasm [47, 48]. Via mutagenesis analysis, five conserved Trp residues (at positions 18, 52, 117, 148, and 183) of LolB were determined to affect membrane localization of *E. coli* lipoproteins [49], and Leu-68 in the protruding loop of LolB was also revealed to play critical roles in the membrane anchoring activity [50]. The *Xcc* LolB protein deduced from the gene contained 218 amino acids, with a typical *N*-terminal lipoprotein signal peptide, and the predicted signal peptidase II cleavage site was at LSG^20^–C^21^V as predicted by signal P software [51]. Conserved domain search showed that it has a LolB domain located at residues 58–214 (bit score: 138.90; E-value: 4.2e-37). The *Xcc* LolB had 25% identity and 41% similarity to *E. coli* LolB (encoded by *b1209* gene of *E. coli* K-12). Sequence analysis displayed that the aforementioned amino acid residues essential for the function of *E. coli* LolB were not fully conserved in *Xcc* LolB. The conserved amino acid residues included Trp-81, Trp-147, and Trp-214, which corresponding to Trp-52, Trp-117, and Trp-183 in *E. coli* LolB. The residues in comparable positions for Trp-18, Leu-68, and Trp-148 in *E. coli* LolB were substituted by Val-39, Val-96, and Ile-179 in Xcc LolB, respectively. The role of these residues in *Xcc* LolB function remains to be elucidated.

Apart from the observations that mutation in the *lolB* gene of *E. coli* affected the localization of lipoproteins, nothing is known about the role of *lolB* in cellular processes of bacteria. Here, we find that *lolB* has multifaceted biological functions in *Xcc*. We first demonstrate that mutation in *lolB* gene affects bacterial attachment of *Xcc* on abiotic surfaces and host leaves (Fig. 1). As biofilm has been characterized as a virulence trait in many phytopathogenic bacteria [52], our phenotypic evaluation showing the involvement of *lolB* in bacterial attachment prompted us to determine whether the *lolB* gene is associated with pathogenicity of *Xcc*. Further, regarding to the general role of biofilm formation in promoting bacterial survival against stresses and protecting bacteria from harsh environment, we reasoned that *lolB* inactivation might impair the growth ability of *Xcc* under stress treatment. Therefore, the roles of *lolB* in pathogenicity, virulence factor production, and stress tolerance were evaluated. We demonstrate that mutation in *lolB* results in a substantial reduction in virulence (Fig. 2). The attenuation in virulence of the *lolB* mutant may result, at least partially if not entirely, from the reduction in extracellular enzyme (including cellulase and mannanase) production (Fig. 3). In addition, the *lolB* mutant grew much slower under heat stress and in the presence of agents that influence integrity of cell membrane compared with that of the wild-type strain (Fig. 4). Because mutation of *lolB* led to reductions in extracellular enzyme production and stress tolerance, it seems reasonable to predict that LolB might influence the expression of genes related to these phenotypes. The reporter assay and RT-qPCR analysis (Fig. 5) revealed that mutation in *lolB* reduced the expression of genes known to be essential for extracellular enzyme production, stress tolerance, and virulence (*engA*, *manA*, *prt1*, *clpP*, and *clpX*). It is implying that *lolB* might affect the expression of these genes transcriptionally and that the decrease in extracellular enzyme production, bacterial attachment, as well as stress tolerance of the *lolB* mutant may be attributable to the reduced transcription of these genes.

Lipoprotein is a crucial structural component of the outer membrane, and is central to the physiology of the Gram-negative cell envelope. It is essential for maintaining cellular integrity, envelope stability, and nutrient acquisition, and also plays roles in bacterial pathogenic mechanisms such as attachment, colonization, and invasion [1, 53]. Bacterial lipoproteins also play an important role in growth and survival of bacteria [54]. Correct localization of lipoproteins is essential for their function, and the Lol system is required for lipoproteins localization [2, 3, 5, 55]. Although the involvement of *Xcc* LolB in lipoprotein localization was not experimentally demonstrated, we discerned that several putative lipoprotein encoding genes were significantly up-regulated in the *lolB* mutant (Table 4) and mutation of *lolB* decreases the integrity of cell membrane (Fig. 6). Hypothetically, we reasoned that the declined biofilm formation attributable to *lolB* inactivation is resulted from changed cell membrane integrity that subsequently affects adhesion ability. It is possible that the *lolB* mutant has impacts in lipoprotein localization and subsequently the outer membrane lipoprotein profile was altered due to *lolB* mutation, thereby sensitizing cells to membrane perturbing compounds such as SDS and EDTA. The findings showing impaired tolerance against heat stress and several membrane-perturbing compounds in *lolB* mutant could be a plausible explanation for the altered membrane integrity seen in *lolB* mutant and suggests that *Xcc* LolB might possess potential role in lipoprotein localization. The mechanism by which the LolB protein acts on lipoprotein outer membrane localization in *Xcc* remains to be experimentally elucidated.

Bacterial lipoproteins contain a characteristic consensus sequence [LVI][ASTVI][GAS]C known as a lipobox [2, 45]. Overall, the putative lipoprotein encoding genes tested in this study showed a typical lipobox (Table 4). None of these putative lipoprotein encoding genes had been characterized with respect to lipid modification and membrane localization. The involvement in *Xcc* attachment, virulence, and stress tolerance of these *lolB* regulated genes is still not known and remains to be explored. It is intriguing that the *lolB* mutant exhibits an increased expression of several putative lipoprotein encoding genes. Together with the above observations, it is suggested that the inactivation of *lolB* might alter the outer membrane lipoprotein profiles and such alterations subsequently stimulate the compensatory pathway(s) to increase the expression of lipoprotein related genes to keep lipoprotein homeostasis. It is also pertinent to note that the *Xcc* LolB possibly affects the transcription of these tested genes indirectly, as the gene product encoded by *lolB* is not belonging to a regulatory protein. LolB likely influences these genes through an unknown regulatory pathway in *Xcc*. Further investigation of the potential genes that encoding the unknown regulatory trail that is activated after *lolB* mutation is needed to confirm the possibility.

## Conclusion

Here, we characterize the *lolB* gene in *Xcc*. By the use of genetic complementation and phenotypic evaluation, we acquired conclusive genetic evidence demonstrated that the *lolB* plays relevant roles in bacterial attachment, extracellular enzyme production, stress tolerance, as well as virulence of *Xcc*. Consistent with phenotypic alterations, the reporter assay and RT-qPCR analysis displayed that the genes encoding major extracellular enzymes, and genes previously reported to be associated with adhesion, stress tolerance, and virulence were reduced in the *lolB* mutant compared with the wild type. To the best of our knowledge, this is the first work to provide insights of the *lolB* physiological roles in multifarious cellular processes, including pathogenicity-related functions and environmental stress adaptation.

## Data Availability

The data generated and/or analyzed during the current study are included in this article.

## Abbreviations

LB: Luria–Bertani
Lol: lipoprotein outer membrane localization
qPCR: quantitative real-time polymerase chain reaction
RT: reverse transcription
*Xcc*: *Xanthomonas campestris* pv. *campestris*
